# L’ulcère de Marjolin: complication rare et tardive des escarres chez un patient paraplégique

**DOI:** 10.11604/pamj.2017.27.117.11140

**Published:** 2017-06-15

**Authors:** Bahija Lemrhari, Soumyia Chiheb

**Affiliations:** 1 Service de Dermatologie-Vénéréologie, CHU Ibn Rochd, Université Hassan II, Casablanca, Maroc

**Keywords:** Ulcère de Marjolin, escarre sacrée, paraplégie, Marjolin’s ulcer, sacred decubitus ulcer, paraplegia

## Image en médecine

L’ulcère de Marjolin désigne la transformation maligne d’une cicatrice de brûlure ou de toute autre plaie ou ulcération chronique. Il est rare, son incidence est estimée à 2% des cas. Le type histologique prédominant reste le carcinome épidermoïde (75%). La survenue d’un carcinome épidermoïde sur une escarre sacrée est une complication exceptionnelle moins de 0,5%; touchant quasi exclusivement les patients paraplégiques et survenant après un délai supérieur à trois décennies (entre blessure et dégénérescence). L’étiologie de cette transformation maligne reste jusqu’à présent inconnue. Le diagnostic est fait souvent tardivement à cause de la chronicité des lésions comme le cas de notre malade. Nous rapportons un cas d’un patient âgé de 64ans, connu paraplégique depuis plusieurs années, suite à un traumatisme du rachis. qui présentait une escarre sacrée sans tendance à la cicatrisation, compliquée d’une lésion ulcéro-bourgennante, macérée, nauséabonde. Une biopsie de la lésion montrait une transformation maligne en faveur d’un carcinome épidermoïde bien différencié infiltrant. Les TDM thoraco-abdomino-pelvienne ne montraient pas d’atteinte osseuse ni d’autres localisations secondaires. Une exérèse large suivie d’une greffe était réalisée. A la lumière de cette observation, la prise en charge correcte des escarres, chez les paraplégiques, est indispensable permettant de prévenir la transformation maligne de ces lésions chroniques.

**Figure 1:**
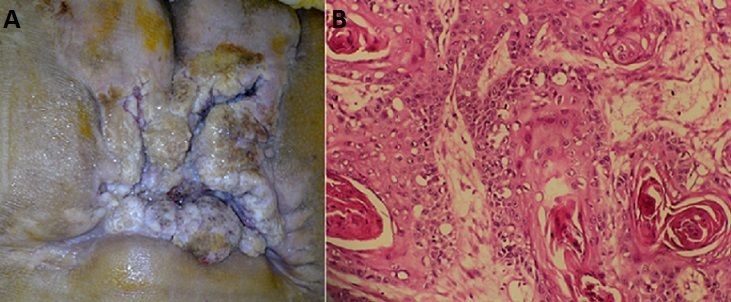
(A) lésion ulcéro-bourgeonnante sur une escarre sacrée; (B) les cellules tumorales sont disposées en boyaux centrés de globes cornées, en faveur de carcinome épidermoide bien différencié

